# Effects of Controlled-Release Nitrogen Fertilizer on Rice Yield, Soil Nutrients, and Nitrogen Use Efficiency in Black Soil with Straw Return

**DOI:** 10.3390/plants15050707

**Published:** 2026-02-26

**Authors:** Yu Zheng, Yue Zhao, Lina Chen, Xingzhu Ma, Xiaoyu Hao, Ying Liu, Jinghong Ji, Shuangquan Liu

**Affiliations:** 1 Heilongjiang Academy of Black Soil Conservation & Utilization, Harbin 150086, China; annadian@163.com (Y.Z.); zhaoyue2108@163.com (Y.Z.); maxingzhu@163.com (X.M.); xiaoyuhao1981@sina.com (X.H.); ly8090@sina.com (Y.L.); 2 Key Laboratory of Black Soil Protection and Utilization, Ministry of Agriculture and Rural Affairs, Harbin 150086, China; 3 Hebei Key Laboratory of Crop Stress Biology, College of Agronomy and Biotechnology, Hebei Normal University of Science and Technology, Qinhuangdao 066000, China; cln3482@hevttc.edu.cn

**Keywords:** black soil, straw return, controlled-release nitrogen fertilizer, rice yield, nitrogen use efficiency

## Abstract

This study used a 3-year field experiment to evaluate the effects of controlled-release nitrogen fertilizers (CRNFs) on rice yield, nitrogen (N) uptake, N recovery efficiency (NRE), N agronomic efficiency (NAE), N partial factor productivity (NPFP), and soil nutrients under straw-returning (SR) conditions in the black soil region of Northeast China. The results showed that CRNF combined with SR increased rice yield, NRE, NAE, and NPFP by 11.2%, 27.7%, 26.1%, and 22.3% respectively; the differences were significant when compared with common N fertilizer (CNF) combined with SR. In addition, CRNF increased soil organic matter (SOM), total N (TN), available N (AN), and other nutrients while reducing nitrate N (NO_3_^−^-N) accumulation in the 30–60 cm soil layer. When the N application rate was reduced by 12%, rice yield still increased by 4.7%, and NRE, NAE, and NPFP increased by 17.2%, 32.9%, and 11.7% respectively; the differences were significant, and the content of soil nutrients has increased to varying degrees. These results indicate that a one-time basal application of controlled-release urea (CRU) mixed with bare urea (BU) at a 6:4 ratio can maintain stable yields, improve fertilization efficiency, reduce N fertilizer input, and lower environmental risks. Therefore, this approach represents an effective strategy for sustainable fertilization in rice-growing areas of Northeast China.

## 1. Introduction

Rice (*Oryza sativa* L.) is an important food crop. About 50% of the world’s population and nearly two-thirds of China’s population rely on rice as a staple food [1]. Northeast China is a major rice-growing region, with an annual planting area of approximately 24.15 × 10^6^ ha, accounting for 20.3% of the national total [2]. The annual rice output is 3.54 × 10^6^ t, representing 17.1% of China’s total production, and it plays a critical role in national food security. However, long-term and intensive application of chemical fertilizers has led to soil quality degradation, which has become a key constraint on sustainable agricultural development [3,4]. In agricultural production, increasing nitrogen (N) fertilizer application is one of the most effective approaches to improve rice yield, contributing to approximately 40% of total grain yield increases [5]. In recent years, yield gains achieved through higher fertilizer inputs have been accompanied by a decline in N use efficiency (NUE). The NUE in China’s rice production is about 35%, which is lower than the global average [6,7]. Therefore, improving N utilization, reducing environmental pollution, and sustaining yield growth have become major research focuses in soil and fertilizer science.

Controlled-release nitrogen fertilizers (CRNFs) regulate nutrient release according to crop growth and development requirements. Compared with conventional fertilizers, CRNFs have a longer effective period, and their nutrient release patterns are generally synchronized with crop nutrient demand. Consequently, CRNFs can meet crop growth needs, increase yield, and improve fertilizer use efficiency [8,9,10]. CRNFs are mainly classified into four types: resin-coated fertilizers, sulfur-coated fertilizers, urea–formaldehyde slow-release fertilizers, and stabilized fertilizers containing biochemical inhibitors [10]. The United States was the earliest country to conduct CRNF research and remains the largest consumer [11]. Sulfur coating was the primary technology in the 1960s [12], while polymer-coating technologies matured in the 1990s. Japan subsequently developed and commercialized resin-, aliphatic polyester-, and paraffin-coated urea fertilizers [13]. Research on CRNFs in China started relatively late but has progressed rapidly, aligning with the goals of low-carbon agriculture and the “zero growth” strategy for chemical fertilizer use. As a result, CRNFs have broad market prospects. Currently, resin-coated urea (CRU) is the most widely used CRNF in agricultural production. Studies have shown that CRNF application can increase rice yield by 6.2–25.3%, reduce fertilizer application rates by 10–40%, and improve NUE by 10–30% [14,15,16]. Khan (2015) reported that sulfur-coated urea combined with urease inhibitors increased maize yield by 38.1%, N uptake by 45.1%, and N recovery efficiency (NRE) by 69.0% [17]. Similarly, Sun et al. [8] demonstrated that, compared with bulk urea (BU), CRU increased rice yield by 11.0%, average NUE by 9.6%, and N agronomic efficiency by 24.97%. Although CRNFs clearly enhance crop yield and fertilizer use efficiency, further research is needed to optimize their performance and improve practical application technologies.

China is a major agricultural country with abundant straw resources. The average annual output of straw from major crops is about 8.64 × 10^8^ tons, accounting for approximately one-fifth of the world’s total straw production [4]. In rural China, straw burning is still common, leading to resource waste, environmental pollution, and damage to soil organic matter (SOM) structure. Crop straw mainly consists of cellulose, hemicellulose, and lignin. Under the action of microorganisms and enzymes, these components decompose and release nutrients, increase soil organic carbon, and thereby enhance soil fertility [18]. Straw return (SR) not only improves soil fertility but also reduces soil bulk density, increases soil porosity and aggregate stability, improves soil physical properties, and enhances sustainable soil productivity [19,20]. Organisms are the basis of biogeochemical cycles and play a key role in soil health and sustainable production [21]. Soil microbial activity and secretions promote rapid straw decomposition, while straw provides an important source of organic matter for microbial metabolism. Soil carbon and N pools are key indicators of soil quality. Soil organic carbon (SOC) and soil organic N (SON) are essential for improving soil fertility and maintaining sustainable agricultural production [22]. Long-term application of organic materials significantly increases SOC and SON, particularly active carbon and N fractions, thereby enhancing soil fertility and promoting healthy crop growth [23]. In recent years, SR has been widely adopted in rice-growing areas of Northeast China as a soil fertility improvement and conservation tillage practice. Numerous studies have reported its positive effects on rice growth and development, yield, fertilizer use efficiency, and soil nutrient availability [24,25,26]. However, studies on the combined effects of SR and N fertilizer management, especially N-reducing and N-free (NRNF) strategies, remain limited, and technical guidance for practical application is still insufficient.

Northeast China is one of the world’s four major black soil belts. Long-term intensive cultivation has led to “thinning, slimming, and hardening” of black soils. “Thinning” refers to a reduction in the depth of topsoil, primarily caused by wind and water erosion; “slimming” denotes a decline in soil nutrient content, mainly resulting from overcropping and unbalanced fertilization; “hardening” describes the deterioration of soil physical properties, largely due to inappropriate tillage practices, long-term omission of organic fertilizer application, and the failure of SR. Reliance on chemical fertilizers alone makes it difficult to significantly increase crop yield and may further accelerate soil degradation and environmental pollution [27]. SR can increase SOM and soil nutrient levels, enhance crop nutrient uptake, reduce chemical fertilizer input, and promote sustainable agricultural development [28]. Northeast China has a cold climate (Figure S1), which slows down the decomposition of straw residues. Consequently, most rice straw is openly burned, leading to environmental pollution and a decline in soil fertility. At present, straw returning is the primary measure adopted in China to solve this issue. Relevant research has mostly focused on the rice–wheat rotation zones and double-cropping rice areas in Southern China. Nevertheless, there are few studies reporting effective techniques for rice straw returning in the high-altitude cold regions of Northeast China, particularly in combination with CRNFs [29]. Many studies have independently examined CRNFs for rice or SR, showing clear effects [30,31]. However, the effects of CRNFs and nitrogen fertilizer management under SR conditions have rarely been studied, especially on black soil in cold regions. Therefore, this study evaluates the effects of CRNFs on rice yield, NUE, and soil nutrient content under SR conditions, aiming to provide technical support and a theoretical basis for efficient rice fertilization and sustainable soil productivity in Northeast China.

## 2. Results

### 2.1. Rice Yield

**Figure 1:**
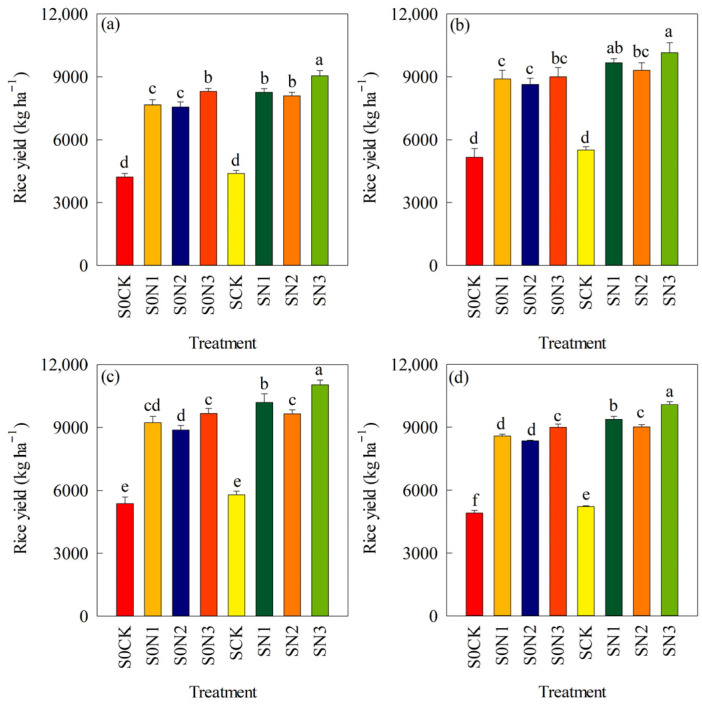
The rice yield with different treatments in different years. (**a**) 2018, (**b**) 2019, (**c**) 2020, (**d**) average value from 2018–2020. Note: S0CK: no SR, no N fertilizer application; S0N1: no SR, 40% BU as basal, 60% BU as top dressing during the tillering stage; S0N2: no SR, 12% N reduction, 40% BU as basal, 60% BU as top dressing during the tillering stage; S0N3: no SR, 12% N reduction, (60% CRU + 40% BU) as basal fertilization without top dressing; SCK: SR, no N fertilizer application; SN1: SR, 40% BU as basal, 60% BU as top dressing during the tillering stage; SN2: SR, 12% N reduction, 40% BU as basal, 60% BU as top dressing during the tillering stage; SN3: SR, 12% N reduction, (60% CRU + 40% BU) as basal fertilization without top dressing. Different lowercase letters indicate significant differences among different treatments (*p* < 0.05).

The experimental results (Figure 1a–c) showed that rice yield differed significantly among SR treatments and different N fertilizer management practices, and similar trends were observed across the 3-year experiment. Based on the average results from 2018 to 2020 (Figure 1d), the yield of S0CK was 4916 kg ha^−1^, compared with this treatment; yields under S0N1, S0N2, and S0N3 increased by 74.7%, 70.0%, and 82.9%, respectively, with significant differences (*p* < 0.05). The yield of SCK was 5227 kg ha^−1^; compared with this treatment, yields under SN1, SN2, and SN3 increased by 79.4%, 72.5%, and 92.7%, respectively, and the differences were significant (*p* < 0.05). Compared with S0CK, S0N1, S0N2, and S0N3, the corresponding treatments with straw returning (SCK, SN1, SN2, and SN3) increased yields by 6.3%, 9.1%, 7.9%, and 12.0%, respectively, with significant differences (*p* < 0.05). The rice yield under the S0N3 treatment increased by 7.6% and 4.7% compared with the S0N2 and S0N1 treatments, respectively (*p* < 0.05), and the rice yield under the SN3 treatment increased by 11.7% and 7.5% compared with the SN2 and SN1 treatments, respectively (*p* < 0.05).

Under conditions without straw returning, the mixed application of CRNF and common N fertilizer (S0N3) increased yield by 7.6% compared with the common N fertilizer treatment (S0N2). Under straw-returning conditions, the average rice yield increased by 11.7% when CRNF was mixed with common N fertilizer and combined with SR (SN3) compared with common N fertilizer combined with SR (SN2). Under a 12% N reduction, the S0N3 treatment increased yield by 4.7% compared with the S0N1 treatment, while under the same N reduction, the SN3 treatment increased yield by 7.5% compared with the SN1 treatment. These results indicate that the application of slow/controlled-release N fertilizers, particularly in combination with straw returning, effectively increases rice yield. Combined application of CRNF and SR improved the yield components of rice, thereby increasing grain yield (Table S1). This integrated application not only enhanced grain yield but also elevated straw yield of rice (Figure S1).

### 2.2. Soil Nutrient

The results (Table 1) showed that SR and different N fertilizer treatments had significant effects on SOM, TN, TP, TK, AN, AP, and AK contents. Compared with the treatment without SR and N application (S0CK), the SR treatment without N application (SCK) significantly increased all soil nutrient indicators, and this trend was consistent across the 3 years. Moreover, the magnitude of the effect increased with experimental duration. Based on the mean values from 2018 to 2020, compared with the S0N2 treatment, the average contents of SOM, TN, TP, TK, AN, AP, and AK in S0N3 increased by 2.8%, 3.6%, 4.5%, 2.7%, 5.1%, 6.8%, and 4.3%, respectively, indicating that CRNF increased soil nutrient contents compared with conventional N fertilizer at the same N rate. Compared with S0N1, the SN1 treatment increased the average contents of SOM, TN, TP, TK, AN, AP, and AK by 4.8%, 12.0%, 6.3%, 11.0%, 24.2%, 14.1%, and 10.0%, respectively. Similarly, compared with S0N2, the SN2 treatment increased these indicators by 5.7%, 9.7%, 7.4%, 14.7%, 25.3%, 13.2%, and 11.6%, respectively. Compared with S0N3, the SN3 treatment increased SOM, TN, TP, TK, AN, AP, and AK by 6.3%, 7.7%, 9.0%, 13.5%, 26.7%, 12.2%, and 12.4%, respectively. Overall, at the same N application rate, SR increased soil nutrient contents to varying degrees compared with straw removal. Under a 12% N reduction, the average contents of SOM, TN, TP, TK, AP, and AK increased by 1.3%, 2.3%, 5.5%, 6.5%, 17.8%, and 6.0%, respectively, when comparing SN3 with SN1. These results indicate that the combination of CRNF and SR promotes soil nutrient accumulation even under a 12% reduction in N application rate.

Table 2 showed that the interaction between the test year (Y) and the contents of SOM, TN, AN, AP, and AK was significant (*p* < 0.05). The interaction between straw returning to the field (S) and the contents of SOM, TN, TP, TK, AN, AP, and AK was extremely significant (*p* < 0.01). The interaction between N fertilizer management (N) and the contents of SOM, TN, TP, TK, AN, and AP was extremely significant (*p* < 0.01). The interaction between Y × S and the contents of SOM, TN, TK, AN, AP, and AK was extremely significant (*p* < 0.01), while that with TP was significant (*p* < 0.05). The interaction between Y × N and the contents of SOM, TN, and AN was significant (*p* < 0.05). The interaction between S × N and the contents of SOM, TN, TK, AN, and AK was significant (*p* < 0.05). The interaction between Y × S × N and the contents of SOM, TN, AN, and AK was significant (*p* < 0.05). These results indicate that the experimental year, straw returning, and N fertilizer management significantly affected soil fertility indicators, and that most interactions among factors reached significant or extremely significant levels.

### 2.3. The Accumulation of NO_3_^−^-N and NH_4_^+^-N in the Soil

**Figure 2:**
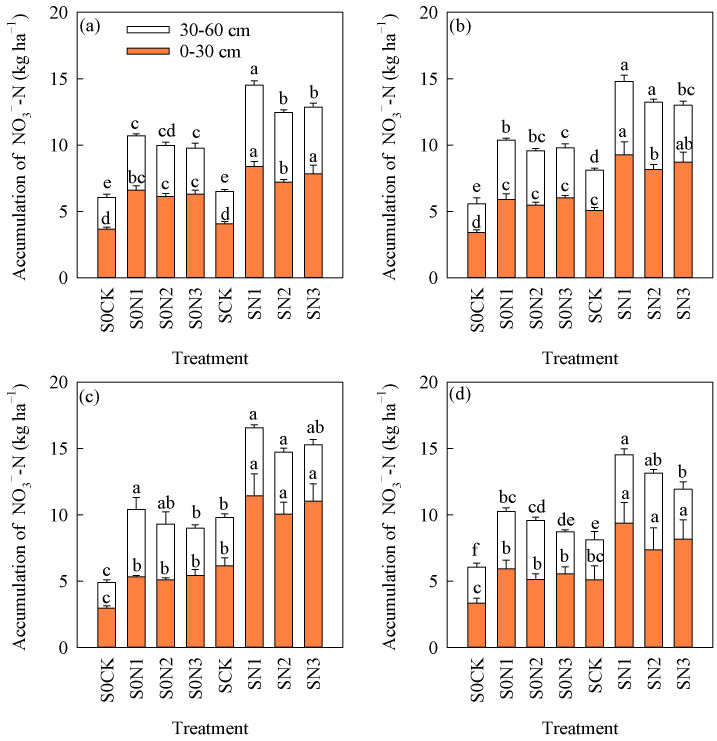
The changes in soil NO_3_^−^-N accumulation in different treatments and different years. (**a**) 2018; (**b**) 2019; (**c**) 2020; (**d**) average value from 2018–2020. Note: S0CK: no SR, no N fertilizer application; S0N1: no SR, 40% BU as basal, 60% BU as top dressing during the tillering stage; S0N2: no SR, 12% N reduction, 40% BU as basal, 60% BU as top dressing during the tillering stage; S0N3: no SR, 12% N reduction, (60% CRU + 40% BU) as basal fertilization without top dressing; SCK: SR, no N fertilizer application; SN1: SR, 40% BU as basal, 60% BU as top dressing during the tillering stage; SN2: SR, 12% N reduction, 40% BU as basal, 60% BU as top dressing during the tillering stage; SN3: SR, 12% N reduction, (60% CRU + 40% BU) as basal fertilization without top dressing. Different lowercase letters indicate significant differences among different treatments (*p* < 0.05).

**Figure 3:**
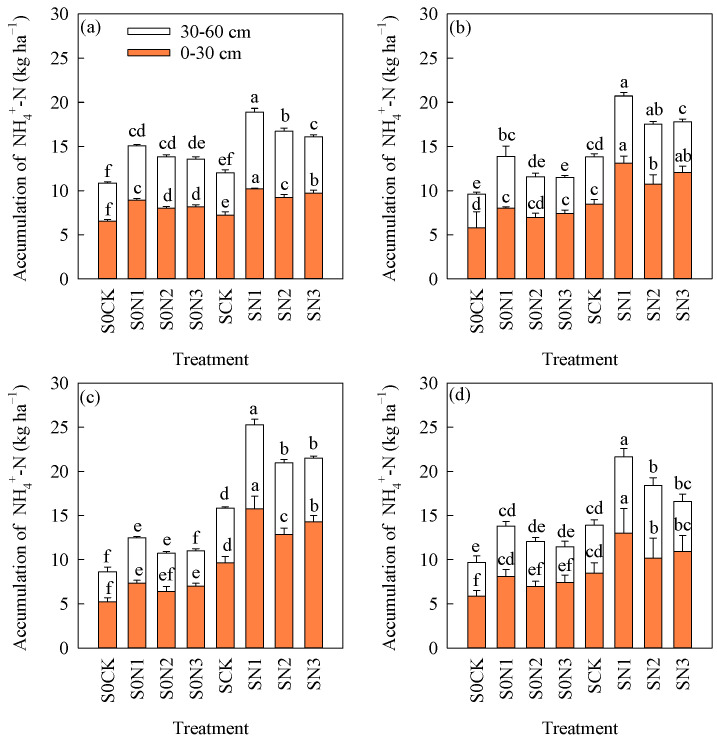
The changes in soil NH_4_^+^-N accumulation in different treatments and different years. (**a**) 2018; (**b**) 2019; (**c**) 2020; (**d**) average value from 2018–2020. Note: S0CK: no SR, no N fertilizer application; S0N1: no SR, 40% BU as basal, 60% BU as top dressing during the tillering stage; S0N2: no SR, 12% N reduction, 40% BU as basal, 60% BU as top dressing during the tillering stage; S0N3: no SR, 12% N reduction, (60% CRU + 40% BU) as basal fertilization without top dressing; SCK: SR, no N fertilizer application; SN1: SR, 40% BU as basal, 60% BU as top dressing during the tillering stage; SN2: SR, 12% N reduction, 40% BU as basal, 60% BU as top dressing during the tillering stage; SN3: SR, 12% N reduction, (60% CRU + 40% BU) as basal fertilization without top dressing. Different lowercase letters indicate significant differences among different treatments (*p* < 0.05).

The experimental results showed (Figure 2 and Figure 3) that straw returning and N fertilizer management significantly affected the accumulation of NO_3_^−^-N and NH_4_^+^-N in the soil, and that these effects became more pronounced with increasing experimental years. The results from 2018 to 2020 showed (Figure 2d) that the average accumulation of NO_3_^−^-N in the soil profile (0–30 cm) increased by 52.2%, 57.7%, 43.8%, and 46.9% under SCK, SN1, SN2, and SN3, respectively, compared with S0CK, S0N1, S0N2, and S0N3. And corresponding the average accumulation of NO_3_^−^-N in the soil profile (30–60 cm) increased by 10.8%, 19.7%, 24.1%, and 27.5%, respectively. The results showed (Figure 3d) that the accumulation of NO_3_^−^-N in the soil profile (0–30 cm) under SCK, SN1, SN2, and SN3 increased by 44.4%, 60.8%, 48.5%, and 47.2%, respectively, compared with S0CK, S0N1, S0N2, and S0N3. The increases in SCK, SN1, SN2, and SN3 compared with S0CK, S0N1, S0N2, and S0N3 were 37.6%, 52.2%, 50.0%, and 42.2% in the soil profile (30–60 cm), respectively. Under SR conditions, the accumulation of NO_3_^−^-N and NH_4_^+^-N in the soil increased to varying degrees compared with treatments without SR.

Under the condition of equal N rate, compared with S0N2, S0N3 increased the accumulation of NO_3_^−^-N and NH_4_^+^-N in the 0–30 cm soil layer by 8.6% and 6.6%, respectively, and decreased their accumulation in the 30–60 cm layer by 10.4% and 7.5%, respectively. Compared with SN2, SN3 increased the accumulation of NO_3_^−^-N and NH_4_^+^-N in the 0–30 cm layer by an average of 10.9% and 7.6%, respectively, while decreasing their accumulation in the 30–60 cm layer by 7.9% and 12.6%, respectively. Under the condition of 12% N reduction, compared with S0N1, S0N3 decreased the accumulation of NO_3_^−^-N and NH_4_^+^-N in the 0–30 cm soil layer by 6.8% and 8.9%, respectively, and in the 30–60 cm layer by 16.7% and 22.8%, respectively. Compared with SN1, SN3 decreased the accumulation of NO_3_^−^-N and NH_4_^+^-N in the 0–30 cm layer by an average of 14.0% and 19.0%, respectively, and in the 30–60 cm layer by 11.3% and 27.9%, respectively (Figure 2d and Figure 3d). As soil depth increased, the accumulation of NO_3_^−^-N and NH_4_^+^-N gradually decreased.

### 2.4. N Uptake in Rice

**Figure 4:**
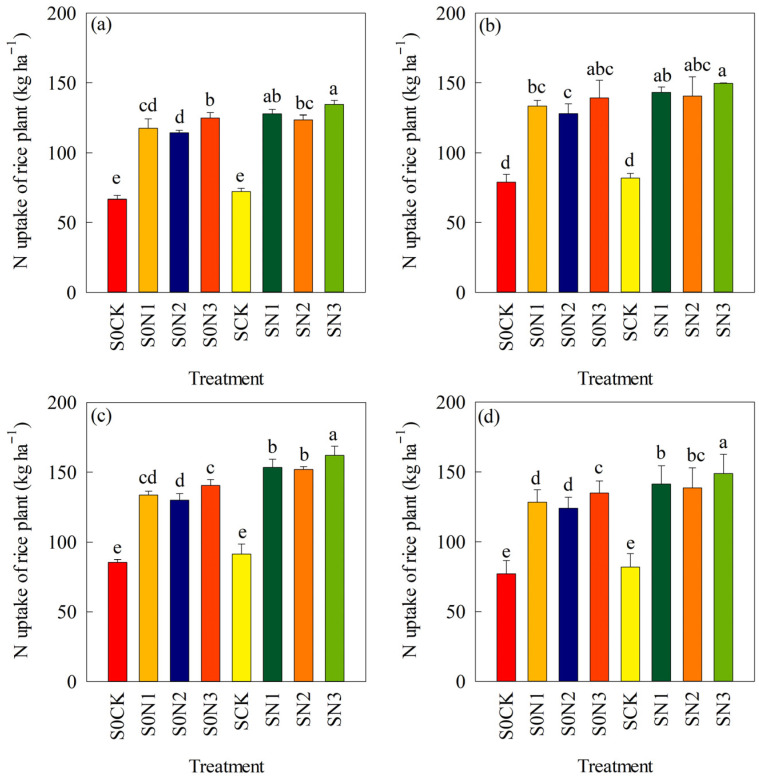
Changes in N uptake of rice in different treatments and different years. (**a**) 2018; (**b**) 2019; (**c**) 2020; (**d**) average value from 2018–2020. Note: S0CK: no SR, no N fertilizer application; S0N1: no SR, 40% BU as basal, 60% BU as top dressing during the tillering stage; S0N2: no SR, 12% N reduction, 40% BU as basal, 60% BU as top dressing during the tillering stage; S0N3: no SR, 12% N reduction, (60% CRU + 40% BU) as basal fertilization without top dressing; SCK: SR, no N fertilizer application; SN1: SR, 40% BU as basal, 60% BU as top dressing during the tillering stage; SN2: SR, 12% N reduction, 40% BU as basal, 60% BU as top dressing during the tillering stage; SN3: SR, 12% N reduction, (60% CRU + 40% BU) as basal fertilization without top dressing. Different lowercase letters indicate significant differences among different treatments (*p* < 0.05).

The experimental results showed (Figure 4) that SR combined with different N fertilizer management strategies significantly promoted N uptake in rice, and the same trend was observed over the 3 years. Compared with S0CK, the average N uptake of rice under S0N1, S0N2, and S0N3 increased by 63.0%, 57.9%, and 75.0%, respectively, with significant differences (*p* < 0.05). Compared with SCK, the average N uptake of rice under SN1, SN2, and SN3 increased by 74.0%, 66.1%, and 84.7%, respectively, with significant differences (*p* < 0.05). Compared with S0CK, S0N1, S0N2, and S0N3, the N uptake of rice under SCK, SN1, SN2, and SN3 increased by 3.1%, 10.1%, 8.5%, and 8.8%, respectively (Figure 4d).

Under the condition of equal N application rate, N uptake in rice under S0N3 increased by an average of 10.9% compared with S0N2, while N uptake under SN3 increased by 11.2% compared with SN2. Under the condition of 12% N reduction, average N uptake under S0N3 increased by 7.4% compared with S0N1, and N uptake under SN3 increased by 8.8% compared with SN1. These results indicate that controlled-release N fertilizer and its combination with straw returning can promote N uptake in rice. However, their effects on the N content of rice straw and grains were not significant (Figures S2 and S3).

### 2.5. N Use Efficiency of Nitrogen Fertilizer

**Figure 5:**
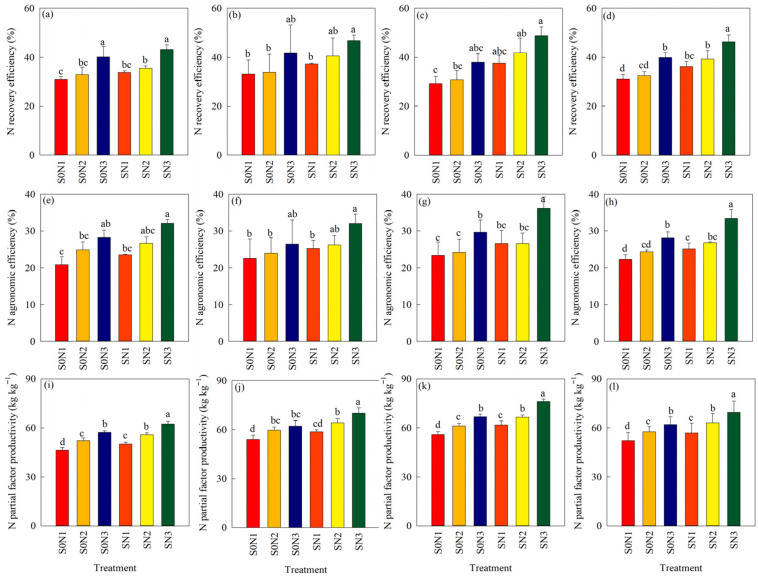
Nitrogen recovery efficiency, agronomic nitrogen efficiency, and nitrogen partial factor productivity of rice under different treatments across different years: (**a**–**d**) 2018, 2019, 2020 and their average for nitrogen recovery efficiency; (**e**–**h**) 2018, 2019, 2020 and their average for agronomic nitrogen efficiency; (**i**–**l**) 2018, 2019, 2020 and their average for nitrogen partial factor productivity. Note: S0CK: no SR, no N fertilizer application; S0N1: no SR, 40% BU as basal, 60% BU as top dressing during the tillering stage; S0N2: no SR, 12% N reduction, 40% BU as basal, 60% BU as top dressing during the tillering stage; S0N3: no SR, 12% N reduction, (60% CRU + 40% BU) as basal fertilization without top dressing; SCK: SR, no N fertilizer application; SN1: SR, 40% BU as basal, 60% BU as top dressing during the tillering stage; SN2: SR, 12% N reduction, 40% BU as basal, 60% BU as top dressing during the tillering stage; SN3: SR, 12% N reduction, (60% CRU + 40% BU) as basal fertilization without top dressing. Different lowercase letters indicate significant differences among different treatments (*p* < 0.05).

The experimental results (Figure 5) show that both N fertilizer management and SR can increase rice NUE, and similar trends were observed over 3 years. NUE increased year by year under CRNF combined with straw returning, whereas NUE decreased year by year under CNF without straw returning. Based on the 3-year average results from 2018 to 2020 (Figure 5d), compared with S0N1, S0N2, and S0N3, the nitrogen recovery efficiency (NRE) of rice under SN1, SN2, and SN3 increased by 16.6%, 20.9%, and 15.7%, respectively, with significant differences (*p* < 0.05). Nitrogen agronomic efficiency (NAE) increased by 12.9% (*p* < 0.05), 8.9%, and 18.9%, respectively, while nitrogen partial factor productivity (NPFP) increased by 4.8%, 4.5%, and 7.5% (*p* < 0.05), respectively.

Under the condition of equal N application rate, the NRE, NAE, and NPFP of rice under S0N3 increased by an average of 28.7%, 15.5%, and 19.1% respectively, compared with S0N2. The NRE, NAE, and NPFP under SN3 increased by an average of 27.7%, 26.1%, and 22.3%, respectively, compared with SN2, with significant differences (*p* < 0.05). Under the condition of 12% N reduction, the NRE, NAE, and NPFP of rice under S0N3 increased by an average of 23.1%, 26.2%, and 7.6%, respectively, compared with S0N1, with significant differences (*p* < 0.05). The NRE, NAE, and NPFP under SN3 increased by 17.2%, 32.9%, and 11.7%, respectively, compared with SN1, with significant differences (*p* < 0.05). These results indicate that CRNF combined with straw returning promotes the improvement of rice NUE in the black soil of this region.

## 3. Discussion

### 3.1. The Influence of N Fertilizer Management and SR on Soil Nutrients

There are many measures in N fertilizer management, including fertilizer type, application rate, application period, and application location [32,33]. Chen et al. [34] demonstrated that when the same amount of CRNF was applied, the contents of AT, AP, and AK in the soil increased by 10.0%, 3.1%, and 4.9% respectively, compared with CNF applied at 225 kg N ha^−1^. This indicates that CRNF application can increase the availability of soil nutrients. The main reason is that CRNF reduces N volatilization and leaching, thereby improving nutrient use efficiency and reducing excessive consumption of soil nutrients by crops [35]. Zhang et al. [36] found that the SOM, TN, and AN content of soil treated with CRNF were higher than those treated with CNF, but CRNF had little effect on TP, TK, AP, and AK contents. The results of this study were generally consistent with these findings. The accumulation and transformation of organic carbon play a key role in regulating soil physicochemical properties and the absorption and release of substances [18]. Straw decomposition produces large amounts of organic carbon, polysaccharides, proteins, and lignin, which promote soil aggregate formation and provide abundant substrates for soil fauna, thereby driving soil carbon and N cycling. In turn, microbial activity promotes straw decomposition [37,38,39]. The results of this study showed that, under SR, all N fertilizer treatments increased soil nutrient contents, N uptake, and NUE compared with N fertilizer application without SR. The combination of SR with CRNF (SN3) increased soil OM, TN, TP, AN, AP, and AK contents compared with CRNF application without SR (S0N3), and these effects became more significant with increasing experimental years (Table 1). Experimental duration, SR, and N fertilizer management were all significantly correlated with SOM, TN, and AN content, while correlations with AP, TK, AP, and AK did not reach consistent significance levels (Table 2). Returning straw to the field increases nutrient enrichment in surface soil and plays an important role in improving soil N, phosphorus, and potassium status. Under conditions of reduced chemical fertilizer application, SR is therefore of great significance for enhancing soil fertility [40,41].

### 3.2. Combination of CRNF and SR on Soil Mineral N Accumulation

NH_4_^+^-N and NO_3_^−^-N in soil are mineral forms of N that plants can directly absorb and utilize and therefore play an important role in rice growth and development. However, excessively high contents, especially high NO_3_^−^-N levels in deep soil layers, pose a serious risk to groundwater safety [42,43,44]. The application of CRNFs increased inorganic N accumulation in the upper soil profile and reduced its accumulation in deeper layers. In the 0–30 cm profile, CRNF increased inorganic N by an average of 12.4% compared with CNF, while in the 60–90 cm profile it decreased inorganic N by an average of 19.7% [9,14]. Straw is the main source of organic N for soil microorganisms, which also promote N transformation and provide N for plant growth. Long-term application of straw to partially replace chemical fertilizer significantly increases soil mineralized N supply capacity and improves soil N supply characteristics [42,43]. Soil bulk density and porosity are the primary indicators for assessing soil structure. Black soils in Northeast China are characterized by high organic matter content and relatively heavy texture. Under normal conditions, the plow layer (0–20 cm) has an organic matter content of 3.76%, a bulk density of 1.26 g·cm^−3^, and a porosity of 52.5%, indicating favorable soil aeration. In contrast, below the plow layer (>20 cm), bulk density increases while porosity decreases, resulting in reduced soil aeration and water permeability [45]. Leaching of NO_3_^−^-N and NH_4_^+^-N is influenced by soil moisture conditions, texture, and other factors, while heavy clayey textures restrict water movement within the soil profile [46]. CRNFs exhibit a gradual nutrient release pattern, and, coupled with the heavy texture of black soils, the application of such fertilizers in black soil regions can effectively reduce the leaching loss of mineral nitrogen into deeper soil profiles (Figure 2 and Figure 3).

Studies have shown that SR increases NH_4_^+^-N and NO_3_^−^-N contents in the 0–20 cm soil layer by 17.1–25.4% and 16.2–36.0%, respectively [26]. Some studies have also reported that, under SR conditions, CRNF reduces soil NH_4_^+^-N and NO_3_^−^-N contents compared with CNF [47].

The results of this study showed that the combination of CRNF and SR increased the accumulation of NO_3_^−^-N and NH_4_^+^-N in the 0–30 cm layer and reduced their accumulation in the 30–60 cm layer under the condition of equal N rate, while under the condition of 12% N reduction, they decreased both in 0–30 cm and 30–60 cm profiles (Figure 2d and Figure 3d). The main reason may be that the continuous and slow N release from CRNF increases crop N uptake and reduces mineral N leaching to deeper soil layers [48]. SR could increase mineral N availability and the combined effects with CRNF enhanced crop N uptake and reduced mineral N leaching losses [26]. As this study was just based on a 3-year experiment, these conclusions require further verification through long-term studies.

### 3.3. The Influence of N Fertilizer Management and SR on Rice Yield

CRNF generally present an S-shaped nutrient release pattern, which closely matches the nutrient uptake pattern of rice and can evenly supply N throughout the growth period [49]. Cui et al. [50] demonstrated that the combination of CRNF and conventional urea increased rice yield by 9.6–18.3% compared with conventional urea. Even when the N application rate was reduced by 20%, yield still increased to some extent [34,51]. This indicates that the multi-level release mechanism of resin-coated controlled-release N fertilizer can meet crop growth requirements and promote yield increase. SR has the functions of increasing soil nutrient availability, improving NUE, and increasing crop yield [52,53]. Studies have shown that under conventional N application levels, rice yield increased by 11.7–17.4% (*p* < 0.05) with SR application, whereas under a 20% N reduction, rice yield with SR showed no significant difference compared with conventional fertilization [54,55]. Some studies have also reported that CRNF and SR do not increase yield [56]. The results of this study showed that S0N3 increased rice yield by 7.6% compared with S0N2, and SN3 increased yield by 11.7% over SN2, while under a 12% N reduction, the average rice yield increased by 7.5% (Figure 1d). The main reason is that the reasonable combination of CRNF and CNF meets the N demand of rice throughout its growth period and improves yield component traits. The average number of effective panicles, grains per panicle, and 1000-grain weight in the CRNF treatment increased by 11.3%, 9.4%, and 2.0%, respectively, compared with the CNF treatment (Table S1). As the experimental years increased, the effect of SR on rice yield enhancement became more pronounced, indicating a cumulative effect. SR provides additional nutrients, while CRNF ensures continuous nutrient supply, and their combined application promotes rice yield increase [57,58].

### 3.4. Nitrogen Management and SR on N Use Efficiency in Rice

Different N fertilizer management models affect N uptake and NUE in rice [59,60]. A reasonable combination of CRNF and CNF can increase N uptake and improve NUE. The main reason is that their combined application can provide a uniform and continuous N supply throughout the rice growth period, thereby promoting growth and reducing N loss [61,62]. Many studies have reported that CRNF promotes N uptake and NUE [63,64]. Chen et al. [65] showed that CRU applied at 216 kg ha^−1^ increased rice NUE by 22.1%, NAE by 44.1%, and NPFP by 28.3% compared with CNF applied at 270 kg N ha^−1^. The results of this study showed that, under equal N rates, CRNF application (S0N3) increased N uptake by 10.9% compared with CNF (S0N2), while average NRE, NAE, and NPFP increased by 23.0%, 15.5%, and 7.6%, respectively. The effect was more pronounced when N input was reduced by 12% (Figure 4 and Figure 5), indicating the positive effect of the combined application of CRNF and CNF on N uptake in rice. Moreover, the increase in N uptake was mainly due to increased grain and straw yields. There was a significant difference in N content between treatments with and without N fertilization, whereas the differences among N-fertilized treatments were small (Figures S2 and S3). Microbial activity is closely correlated with ambient temperature. During the early growth stage of rice in the cold regions of Northeast China, the average temperature is merely around 20 °C, leading to relatively low microbial activity [66]. Consequently, straw decomposition proceeds slowly, which triggers a nitrogen (N) competition between soil microbes and rice plants; this inhibits rice growth and N uptake to a certain extent. By contrast, the sustained slow release of N from CRNFs mitigates this limitation. As temperatures rise, straw decomposition accelerates, continuously releasing nitrogen nutrients, facilitating rice N uptake, and promoting dry matter accumulation. SR increases the quantity and activity of soil microorganisms, promotes soil N transformation, enhances soil N supply capacity, facilitates crop N uptake, and improves fertilizer use efficiency [58,67]. Compared with CNF alone, CNF combined with SR increased rice NRE, NAE, and NPFP by 10.8–19.1%, 61.1–78.6%, and 19.5–26.5%, respectively [54,68]. The results of this study showed that CRNF combined with SR (SN3) increased rice N uptake, NRE, NAE, and NPFP by 11.2%, 27.7%, 26.1%, and 22.3%, respectively, compared with CNF combined with SR (SN2) (Figure 4 and Figure 5). The combination of CRNF and SR improved rice NUE [69], which may be attributed to the reasonable CRNF and CNF ratio (6:4) that better meets rice N demand throughout the growth period. In addition, SR increases the supply and retention of available soil N, promotes N uptake, and thereby improves rice NUE. The increase in N uptake was mainly due to increased rice biomass (Figure S1). There was little difference in N content in rice grains and straw among N-fertilized treatments at maturity stage (Figures S2 and S3).

## 4. Materials and Methods

### 4.1. Overview of the Experimental Site

The trial began in April 2018 and ended in December 2020, lasting for 3 years. The experiment was conducted at the experimental site of Fangzheng County Agricultural Technology Extension Center in Harbin City, Heilongjiang Province (45°51′56.0″ N, 128°48′23.6″ E), and the soil type was black soil. Basic physical and chemical properties of 0–20 cm soil: soil organic matter (SOM) 31.89 g kg^−1^, total nitrogen (TN) 1.67 g kg^−1^, total phosphorus (TP) 1.13 g kg^−1^, total potassium (TK) 28.67 g kg^−1^, available nitrogen (AN) 158.6 mg kg^−1^, available phosphorus (AP) 40.4 mg kg^−1^, available potassium (AK) 173.3 mg kg^−1^, and pH 6.61. Fangzheng County is located in the central and southern part of Heilongjiang Province. It belongs to cold-temperate continental monsoon climate with an average annual temperature of 2.6 °C, a frost-free period of about 130 days, an annual accumulated temperature (≥10 °C) of 2400–2700 °C, and an annual precipitation of 500–700 mm, where the rain and heat are of the same intensity, so is suitable for rice growth [70]. The annual precipitation in 2018, 2019, and 2020 stood at 598.1 mm, 613.0 mm, and 876.3 mm, respectively, while the effective accumulated temperature (≥10 °C) over the same period was about 2400–2500 °C, 2500–2600 °C, and 2600–2700 °C in the corresponding years. Interannual temperature variations for 2018, 2019, and 2020 are presented in Figure S4.

### 4.2. Experimental Design

A split-zone test design was adopted. The main zone was SR, with two treatments: S0 and S. The sub-zone was N fertilizer management, with three treatments (N1, N2, and N3) and two controls. In total, eight treatments were included: (1) S0CK, no SR, and no N fertilizer; (2) S0N1, no SR, 40% BU applied as basal fertilizer, and 60% BU applied as top dressing at the tillering stage; (3) S0N2, no SR, 12% N reduction, 40% BU as basal fertilizer, and 60% BU as top dressing at the tillering stage; (4) S0N3, no SR, 12% N reduction, with 60% CRU + 40% BU applied as basal fertilizer without top dressing; (5) SCK, SR with no N fertilizer; (6) SN1, SR, 40% BU as basal fertilizer, and 60% BU atop dressing at the tillering stage; (7) SN2, SR, 12% N reduction, 40% BU as basal fertilizer, and 60% BU as top dressing at the tillering stage; and (8) SN3, SR, 12% N reduction, with 60% CRU + 40% BU applied as basal fertilizer without top dressing. Based on years of multi-site trials with various CRU:BU ratios, the 6:4 mixing ratio of CRU to BU was selected as the optimal ratio for local rice production [14,71]. The main plot area was 60 m^2^ (12 m × 5 m), and the sub-plot area was 20 m^2^ (4 m × 5 m) with three replications. In autumn, rice straw was collected from adjacent experimental plots, crushed into 3–5 cm segments, weighed in accordance with the experimental requirements for SR, and incorporated into the soil prior to plowing. The rice variety used was Suijing 18. Bare urea (BU, N 46%, CNPC Daqing Petrochemical, Daqing, China) was used as the conventional N fertilizer, and resin-coated controlled-release urea (CRU, N 44%, Shandong Kingenta Fertilizer Group Co., Ltd., Linyi , China) was used as the CRNF. Triple superphosphate (P_2_O_5_ 46%, Guizhou Phosphate Group, Guiyang, China) and potassium chloride (KCl 60%, Qinghai Salt Lake Potash Co., Ltd., Golmud, China) were used as phosphorus and potassium fertilizers, respectively; the experimental treatments are shown in Table 3.

### 4.3. Soil and Plant Sampling and Analysis

#### 4.3.1. Soil Sampling and Analysis

Plots were established according to the experimental design. Before the experiment, soil samples from the 0–20 cm layer were collected as baseline samples. After harvest each autumn, soil samples were collected from the 0–30 cm and 30–60 cm layers of each plot. Five sampling points were selected per plot, and one composite sample was prepared for each depth, resulting in 48 samples per year. Soil samples were transported to the laboratory in cooling boxes for nutrient analysis. The fresh soil samples were extracted with 0.01 M CaCl_2_ to determine mineral N (NO_3_^−^-N and NH_4_^+^-N) using an auto-analyzer [72] (Model AA3-A001–02E, Bran-Luebbe, Norderstedt, Germany). The remaining samples were air-dried, ground, passed through a 0.25 mm sieve, and stored in paper bags for further nutrient analysis.

The soil pH was determined by the potentiometric method using a water-to-soil ratio of 2.5:1. SOM was measured using the K_2_CrO_7_ external heating method. Total N (TN) was determined by concentrated H_2_SO_4_-HNO_3_ digestion followed by analysis with a Kjeldahl N analyzer (K9840, Hanon, Jinan, China). Total phosphorus (TP) was determined through digestion with concentrated H_2_SO_4_-HClO_4_ and the molybdenum–antimony colorimetric method. Total potassium (TK) was measured through NaOH fusion and atomic absorption spectrophotometry (TAS-990, PERSEE, Beijing, China). Available N (AN) was determined using the alkali-hydrolyzed diffusion method. Available phosphorus (AP) was extracted with 0.5 M NaHCO_3_ and determined through the molybdenum–antimony colorimetric method. Available potassium (AK) was extracted with NH_4_OAc and determined through atomic absorption spectrometry [73].

#### 4.3.2. Plant Sampling and Analysis

The rice plants and grain samples were collected after harvest. In the laboratory, plant samples were oven-heated at 105 °C for 30 min to deactivate enzymes, then dried at 75 °C to constant weight, weighed, and ground to pass through a 0.5 mm sieve. The total plant N concentration was determined through H_2_SO_4_-H_2_O_2_ digestion followed by the micro-Kjeldahl method [73].

### 4.4. The Method of Calculation

The N uptake of rice and NUE calculated as follows [74]:

N uptake (kg ha^−1^) = grain yield × N content (%) + straw yield × N content (%) (1)

NRE (%) = (Nf − Nu)/Na × 100(2)

Nf is the N uptake (grain plus straw) of the fertilized plot (kg), Nu is the N uptake (grain plus straw) of the unfertilized plot (kg), and Na is the quantity of N applied (kg).

NAE (kg kg^−1^) = (GYf − GYu)/Na × 100 (3)

GYf is the grain yield of the fertilized plot (kg), GYu is the grain yield of the unfertilized plot (kg), and Na is the quantity of N applied as nitrogen fertilizer (kg); the same applies to Equation (4).

NPFP (kg kg^−1^) = GYf/Na (4)

The soil inorganic N accumulation was calculated as follows [75,76]:

NO_3_^−^-N accumulation (kg ha^−1^) = soil depth (cm) × soil bulk density (g cm^−3^) × NO_3_^−^-N content (mg kg^−1^)/10 (5)

NH_4_^+^-N accumulation (kg ha^−1^) = soil depth (cm) × soil bulk density (g cm^−3^) × NH_4_^+^-N content (mg kg^−1^)/10 (6)

### 4.5. Data Processing and Statistical Analysis

The normality and homogeneity of variances of all datasets were verified via the Shapiro–Wilk test prior to performing ANOVA and LSD tests. Multivariate analysis of variance (ANOVA) was performed using IBM SPSS Statistics 27 (SPSS, Chicago, IL, USA) based on the general c model univariate procedure. The effects of experimental year, SR, and N management on SOM, TN, TP, TK, AN, AP, and AK were evaluated using two-way ANOVA. Differences among factor levels were compared using the least significant difference (LSD) test (*p* < 0.05). The effects of treatments on rice grain yield, N uptake, N use efficiency, and soil properties were analyzed through two-way ANOVA using the DPS 18.10 software (Hangzhou Ruifeng Information Technology Co., Hangzhou, China), followed by the LSD test (*p* < 0.05). Graphs were generated using SigmaPlot 14.0 (Systat Software, Inc., San Jose, CA, USA).

## 5. Conclusions

This paper investigated the effects of different N fertilizer management strategies on rice yield, NUE, and soil nutrients in black soil under SR through 3 years of field experiments. The results showed that under SR conditions, all N fertilizer treatments (SN1, SN2, and SN3) increased rice yield, N uptake, NUE, and soil nutrient contents to varying degrees compared with the corresponding treatments without SR (S0N1, S0N2, and S0N3), with SN3 performing best. At the same N application rate, the combination of CRNF, CNF and SR (SN3) increased rice yield, N uptake, and NUE compared with SN2. In addition, SN3 increased soil OM, TN, TP, TK, AN, AP, and TK contents while reducing NO_3_^−^-N and NH_4_^+^-N in the 30–60cm soil layer. Under a 12% N reduction, CRNF combined with SR (SN3) still improved rice yield, N uptake, NUE, and soil nutrient status compared with CNF combined with SR (SN1). These results indicate that under SR conditions, applying a mixture of CRNF and CNF at a 6:4 ratio as a single basal fertilizer (SN3) is feasible and can provide technical and theoretical support for efficient rice fertilization and sustainable soil productivity in Northeast China. The experimental results showed that the nitrogen fertilizer application rate was reduced by 12% only under straw incorporation and when CRNF and CNF were mixed at a 6:4 ratio and applied as a one-time basal application. Future studies should focus on the interaction mechanisms between slow- and controlled-release N fertilizers and straw returning, as well as on establishing long-term fixed-field monitoring and experimental platforms.

## Figures and Tables

**Table 1:** Soil nutrients under different treatments and in different years.

Year	Treat.	SOM (g kg^−1^)	TN (g kg^−1^)	TP (g kg^−1^)	TK (g kg^−1^)	AN (mg kg^−1^)	AP (mg kg^−1^)	AK (mg kg^−1^)
2018	S0CK	31.5 b	1.38 d	0.97 b	27.3 c	153.9 c	36.1 c	152.5 f
	S0N1	32.7 a	1.58 ab	1.17 ab	28.8 bc	169.1 ab	40.1 b	158.1 ef
	S0N2	32.2 a	1.49 bcd	1.20 a	29.0 abc	157.2 bc	43.9 ab	164.7 cde
	S0N3	32.6 a	1.55 ab	1.25 a	29.8 ab	163.8 abc	45.5 ab	170.6 abc
	SCK	32.1 a	1.41 cd	1.09 ab	28.1 bc	157.3 bc	37.9 c	159.1 def
	SN1	33.4 a	1.63 a	1.23 a	29.7 ab	171.9 a	41.9 b	167.6 bcd
	SN2	32.6 a	1.53 abc	1.24 a	30.3 ab	160.9 abc	46.3 a	174.0 ab
	SN3	33.1 a	1.58 ab	1.28 a	31.2 a	167.2 a	48.2 a	178.1 a
2019	S0CK	30.3 b	1.29 d	0.89 c	26.5 c	145.2 c	32.1 e	147.9 d
	S0N1	32.2 ab	1.52 abc	1.17 ab	28.1 abc	160.1 abc	35.9 de	153.6 cd
	S0N2	31.7 ab	1.41 cd	1.22 ab	28.4 abc	144.0 c	37.6 de	156.8 cd
	S0N3	32.1 ab	1.45 bc	1.26 ab	29.1 abc	153.9 ab	41.2 cd	165.6 bc
	SCK	32.3 b	1.47 bc	1.13 b	27.5 bc	161.4 ab	40.3 cd	163.3 bc
	SN1	34.0 ab	1.62 a	1.27 ab	29.1 abc	178.6 a	44.6 bc	175.5 ab
	SN2	32.9 ab	1.58 ab	1.32 a	29.8 ab	166.3 ab	48.2 ab	179.2 a
	SN3	33.7 a	1.64 a	1.34 a	30.3 a	172.5 a	51.4 a	185.9 a
2020	S0CK	29.0 c	1.24 c	0.79 e	25.9 e	137.5 e	27.2 e	143.4 d
	S0N1	31.5 abc	1.41 b	1.08 d	27.6 de	152.8 cd	30.9 de	147.4 d
	S0N2	29.7 bc	1.32 bc	1.17 cd	28.0 e	136.2 e	34.1 d	153.6 cd
	S0N3	31.1 abc	1.37 bc	1.24 bcd	28.8 de	141.8 de	36.8 d	159.1 bc
	SCK	32.7 abc	1.58 a	1.21 cd	28.3 de	164.7 bc	42.9 c	168.6 b
	SN1	34.3 a	1.71 a	1.33 abc	30.9 cd	184.7 a	46.3 bc	180.9 a
	SN2	33.4 ab	1.65 a	1.38 ab	31.6 bc	174.6 ab	50.4 b	184.7 a
	SN3	34.7 a	1.69 a	1.42 a	34.1 a	181.9 a	56.9 a	191.6 a
Aver.	S0CK	30.3 d	1.30 e	0.88 d	26.6 e	145.5 d	31.8 e	147.9 e
	S0N1	32.1 bc	1.50 bcd	1.14 c	28.2 de	160.7 bcd	35.6 de	153.0 de
	S0N2	31.2 cd	1.41 de	1.20 bc	28.5 cd	145.8 d	38.5 cde	158.3 cde
	S0N3	32.1 bc	1.46 cd	1.25 abc	29.2 bcd	153.2 cd	41.2 bcd	165.1 bc
	SCK	32.4 bc	1.49 cd	1.14 c	28.0 de	161.1 bcd	40.4 cd	163.7 bcd
	SN1	33.6 ab	1.65 a	1.28 ab	29.9 bc	178.4 a	44.3 bc	174.7 ab
	SN2	33.0 ab	1.57 abc	1.31 a	30.6 ab	167.3 abc	48.3 ab	179.3 a
	SN3	34.1 a	1.64 ab	1.35 a	31.9 a	173.9 ab	52.2 a	185.2 a

Note: Data (mean, *n* = 3) with different letters after them indicate a statistically significant difference based on LSD (*p* < 0.05). SOM: organic matter; TN: total nitrogen; TP: total phosphorus; TK: total potassium: AN: available nitrogen; AP: available phosphorus; AK: available potassium. S0CK: no SR, no N fertilizer application; S0N1: no SR, 40% BU as basal, 60% BU as top dressing during the tillering stage; S0N2: no SR, 12% N reduction, 40% BU as basal, 60% BU as top dressing during the tillering stage; S0N3: no SR, 12% N reduction, (60% CRU + 40% BU) as basal fertilization without top dressing; SCK: SR, no N fertilizer application; SN1: SR, 40% BU as basal, 60% BU as top dressing during the tillering stage; SN2: SR, 12% N reduction, 40% BU as basal, 60% BU as top dressing during the tillering stage; SN3: SR, 12% N reduction, (60% CRU + 40% BU) as basal fertilization without top dressing.

**Table 2:** The analysis of the interaction between experimental factors and soil nutrients.

Factor	SOM	TN	TP	TK	AN	AP	AK
Year (Y)	*	*	ns	*	*	*	*
Straw (S)	**	**	**	**	**	**	**
N management (N)	**	**	**	**	**	**	ns
Y × S	**	**	*	**	**	**	**
Y × N	*	*	ns	ns	*	ns	ns
S × N	*	*	ns	*	*	ns	*
Y × S × N	*	*	ns	ns	*	ns *	*

Note: * and ** indicate significant difference at the 0.05 and 0.01 levels, respectively, and ns indicates no significant difference. SOM: organic matter; TN: total nitrogen; TP: total phosphorus; TK: total potassium: AN: available nitrogen; AP: available phosphorus; AK: available potassium.

**Table 3:** Experimental treatment and nutrient dosage (kg ha^−1^).

Treatment	N	P_2_O_5_	K_2_O	Straw
S0CK	0	65	82.5	0
S0N1	165 (100% BU)	65	82.5	0
S0N2	145 (100% BU)	65	82.5	0
S0N3	145 (40% BU + 60% CRU)	65	82.5	0
SCK	0	65	82.5	6500
SN1	165 (100% BU)	65	82.5	6500
SN2	145 (100% BU))	65	82.5	6500
SN3	145 (40% BU + 60% CRU)	65	82.5	6500

Note: S0CK: no SR, no N fertilizer application; S0N1: no SR, 40% BU as basal, 60% BU as top dressing during the tillering stage; S0N2: no SR, 12% N reduction, 40% BU as basal, 60% BU as top dressing during the tillering stage; S0N3: no SR, 12% N reduction, (60% CRU + 40% BU) as basal fertilization without top dressing; SCK: SR, no N fertilizer application; SN1: SR, 40% BU as basal, 60% BU as top dressing during the tillering stage; SN2: SR, 12% N reduction, 40% BU as basal, 60% BU as top dressing during the tillering stage; SN3: SR, 12% N reduction, (60% CRU + 40% BU) as basal fertilization without top dressing.

## Data Availability

The data presented in this study are available on request from the corresponding authors due to related scientific research and application needs.

## Supplementary Materials

The following supporting information can be downloaded at: https://www.mdpi.com/article/10.3390/plants15050707/s1, Table S1: Rice yield components of different treatment in different years; Figure S1: The straw yield of rice with different treatments in different years; Figure S2: The nitrogen content in rice grain of different treatment in different year; Figure S3: The nitrogen content in rice straw of different treatment in different years; Figure S4: The variation in air temperature 2018–2020.
