# Supplementary material for: Effects of Controlled-Release Nitrogen Fertilizer on Rice Yield, Soil Nutrients, and Nitrogen Use Efficiency in Black Soil with Straw Return
Source: Plants (Basel). 2026 Feb 26;15(5):707. doi: 10.3390/plants15050707 (PMC12986997; doi:10.3390/plants15050707)
Supplement: Supplementary file 1 [file plants-15-00707-s001.zip › plants-4107157-supplementary.pdf]

Table S1 Rice yield components of different treatment in different years.

Treatment	2018			2019			2020			2018–2020		
	No. of effective panicle (m^{-1})	No. of grains per panicle	1000-weight (g)	No. of effective panicle (m^{-1})	No. of grains per panicle	1000-weight (g)	No. of effective panicle (m^{-1})	No. of grains per panicle	1000-weight (g)	No. of effective panicle (m^{-1})	No. of grains per panicle	1000-weight (g)
S0CK	219.0e	69.2c	24.4b	299.9d	73.9f	23.9e	327.4d	88.9c	24.3d	299.9c	71.3d	23.9d
S0N1	329.0cd	86.8ab	24.8b	410.5d	97.2c	25.4bc	423.6abc	106.9abc	26.1abc	410.5b	90.5abc	25.4b
S0N2	302.1d	74.1bc	25.2ab	401.0b	89.3d	25.0c	387.5bcd	103.3abc	25.2bcd	401.0b	83.2bc	25.0bc
S0N3	377.4ab	83.1bc	26.4a	438.0b	101.8b	26.3ab	408.6abc	116.7ab	26.5ab	424.5ab	92.9ab	26.3a
SCK	390.6a	84.1abc	24.9b	335.7c	85.7e	24.5d	367.7cd	100.1bc	24.7cd	379.3c	80.1cd	24.5cd
SN1	338.3bcd	99.5 a	25.7ab	441.0a	108.2ab	26.2ab	453.8ab	116.1ab	26.8a	441.0ab	99.2a	26.2a
SN2	357.4abc	78.3bc	25.4ab	437.0a	94.6c	25.3c	432.2abc	108.8ab	25.5bcd	437.0ab	87.5abc	25.3b
SN3	364.1abc	84.1abc	26.7a	466.0a	106.0a	26.7a	467.1a	121.6a	26.9a	457.2a	96.3a	26.7a

Note: S0CK: no SR, no N fertilizer application; S0N1: no SR, 40% BU as basal, 60% BU as top dressing during the tillering stage; S0N2: no SR, 12% N reduction, 40% BU as basal, 60% BU as top dressing during the tillering stage; S0N3: no SR, 12% N reduction, (60% CRU + 40%BU) as basal fertilization without topdressing; SCK: SR, no N fertilizer application; SN1: SR, 40% BU as basal, 60% BU as top dressing during the tillering stage; SN2: SR, 12% N reduction, 40% BU as basal, 60% BU as top dressing during the tillering stage; SN3: SR, 12% N reduction, (60% CRU + 40%BU) as basal fertilization without top dressing. Different lowercase letters indicate significant differences among different treatments ($p < 0.05$).

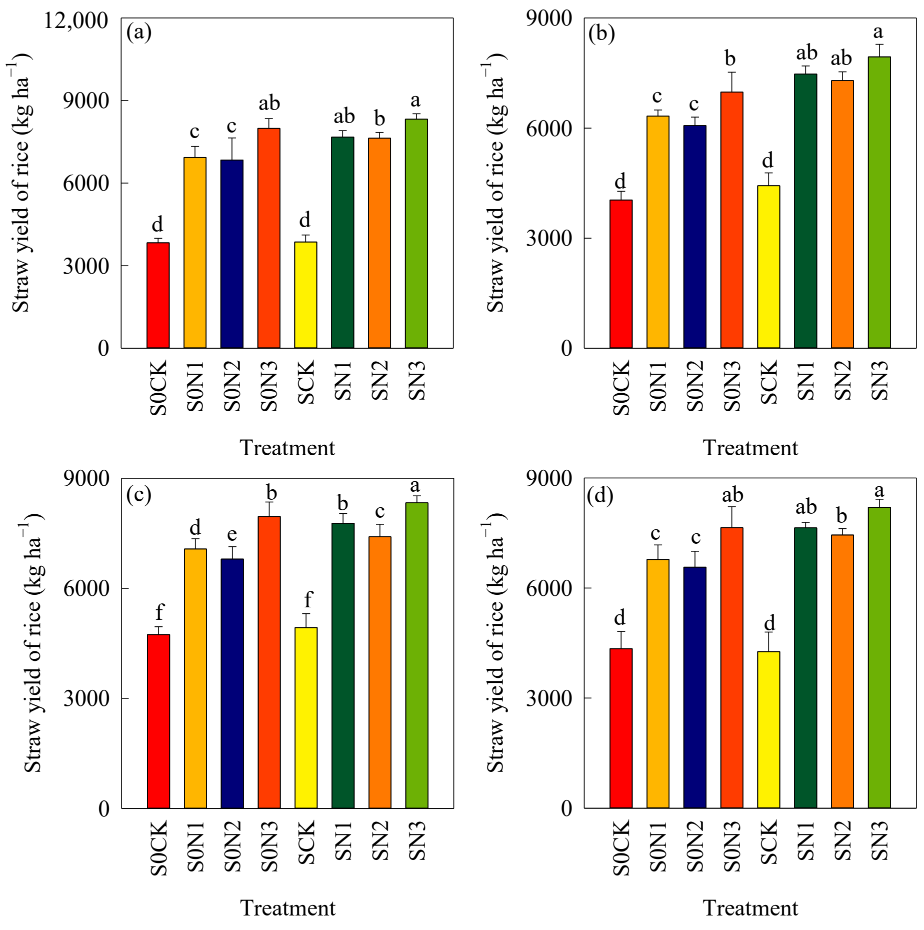

Figure S1 The straw yield of rice with different treatments in different years

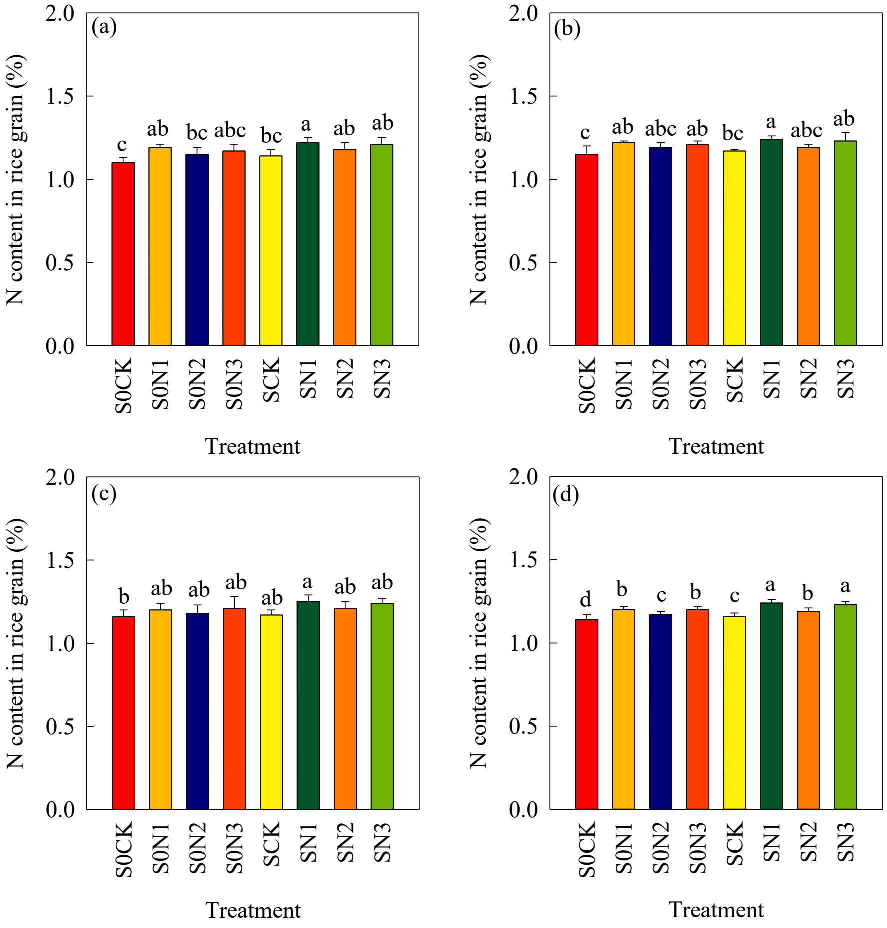

Figure S2 The nitrogen content in rice grain of different treatment in different years

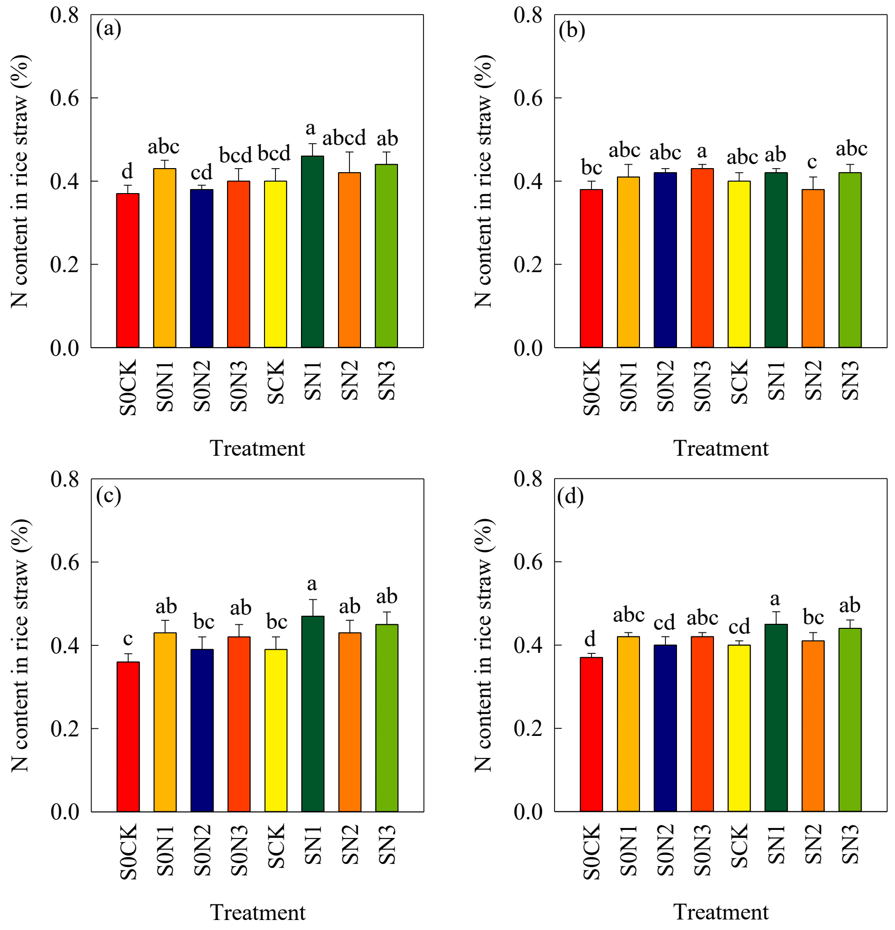

Figure S3 The nitrogen content in rice straw of different treatment in different years

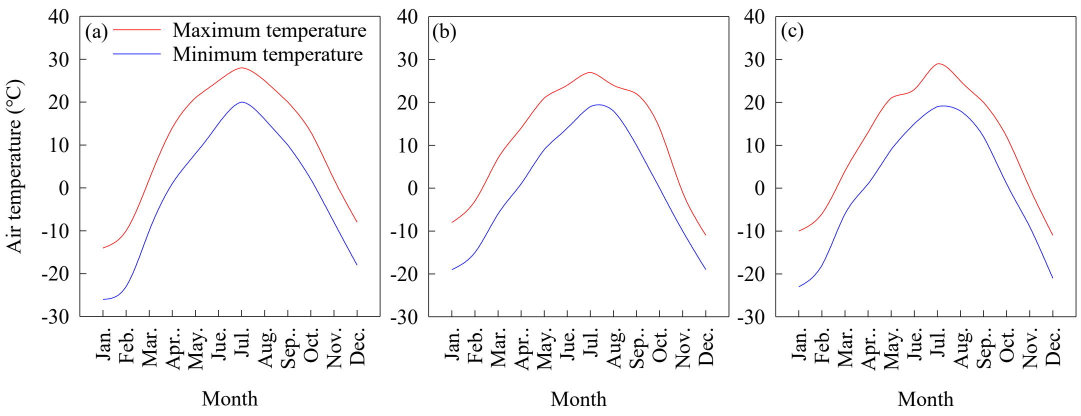

Figure S4 The variation of air temperature during 2018–2020.
